# Impact of daily soft‐tissue image guidance to prostate on pelvic lymph node (PLN) irradiation for prostate patients receiving SBRT

**DOI:** 10.1002/acm2.12665

**Published:** 2019-06-17

**Authors:** Neelam Tyagi, Elizabeth Hipp, Michelle Cloutier, Tomer Charas, Sandra Fontenla, James Mechalakos, Margie Hunt, Michael Zelefsky

**Affiliations:** ^1^ Department of Medical Physics Memorial Sloan Kettering Cancer Center New York NY USA; ^2^ Department of Radiation Oncology Memorial Sloan Kettering Cancer Center New York NY USA

**Keywords:** image guidance, pelvic lymph node, prostate cancer, stereotactic body radiation therapy

## Abstract

**Purpose:**

To determine the impact of using fiducial match for daily image‐guidance on pelvic lymph node (PLN) coverage for prostate cancer patients receiving stereotactic body radiation therapy (SBRT).

**Methods:**

Thirty patients underwent SBRT treatment to the prostate and PLN from 2014 to 2016. Each patient received either 800cGy × 5 or 500cGy × 5 to the prostate and 500cGy × 5 to the PLN. A 5 mm clinical target volume (CTV)‐to‐planning target volume (PTV) margin around the PLN was used for planning. Two registrations with planning computed tomography (PCT) for each of the daily cone beam CTs (CBCTs) were performed: a rigid registration to fiducials and to the bony anatomy. The average translational difference between fiducial and bony match as well as percentage of fractions with differences > 5mm were calculated. Changes in bladder and rectal volume as well as center‐of‐mass (COM) position from simulation parameters, and their correlation with translational difference were also evaluated. The dosimetric impact of the translational differences was calculated by shifting the plan isocenter.

**Results:**

The average translational difference between fiducial and bony match was 0.06 ± 0.82, 2.1 ± 4.1, −2.8 ± 4.3, and 5.5 ± 4.2 mm for lateral, vertical, longitudinal, and vector directions. The average change in bladder and rectal volume from simulation was −67.2 ± 163.04 cc (−12 ± 52%) and −1.6 ± 18.75 (−2 ± 30%) cc. The average change in COM of bladder from the simulation position was 0.34 ± 2.49, 4.4 ± 8.1, and −3.9 ± 7.5 mm along the LR, AP, and SI directions. The corresponding COM change for the rectum was 0.17 ± 1.9, 1.34 ± 3.5, and −0.6 ± 5.2 mm.

**Conclusions:**

The 5 mm margin covered ~75% of fractions receiving PLN irradiation with SBRT, daily CBCT and fiducial‐guided setup. The dosimetric impact on PLN coverage was significant in 19% of fractions or 25% of patients. A larger translational shift was due to variation in rectal volume and changes in COM position of the bladder and rectum. A consistent bladder positioning and/or rectum filling compared with presimulation volume were essential for adequate coverage of PLN in a hypofractionated treatment regime.

## 1 INTRODUCTION

Radiotherapy (RT) is a commonly used modality for the treatment of prostate cancer. Patients with high‐risk prostate cancer, which is defined by the National Comprehensive Cancer Network as having at least one adverse feature (T3a or higher; Gleason score 8–10, or prostate‐specific antigen > 20 ng/mL), have an increased risk for nodal involvement.^1^ Therefore, it is recommended that pelvic lymph nodes (PLN) be included in the target volume for those patients. In addition, there is evidence of clinical benefit from PLN treatment in the high‐risk population.^2^, ^3^ Given the low α/β ratio attributed to prostate cancer, (approximately 1.5–1.8), hypofractionation is potentially more beneficial for occult nodal metastases in high‐risk patients.^4^ At our institution, high‐risk prostate patients with Gleason score > 8 are considered to undergo PLN external beam irradiation. Because more accelerated courses of moderate and ultra‐hypofractionation RT are currently being used for prostate treatments, evaluating the role of PLN treatment with hypofractionation continues. The ongoing phase II SATURN trial (NCT01953055), assessing the toxicity and clinical outcomes of 500 cGy per fraction to pelvic nodes, has finished accrual; a report of the initial outcomes is expected in 2019. Several small, preliminary trials have been reported, evaluating the feasibility and safety of this approach.^5^, ^6^, ^7^, ^8^ At our institution, hypofractionated PLN RT is done in specific cases, and the urinary and bowel toxicity of 21 patients treated with PLN hypofractionation was recently evaluated. At this point, with a median follow‐up of 9 months, urinary and gastrointestinal toxicities were evaluated, which will be reported in a future study. Preliminary assessments indicate that stereotactic body radiation therapy (SBRT) to PLNs given in five fractions (for a total dose of 25 Gy) using dose painting to the prostate and seminal vesicles is safe and well tolerated without increased rates of gastrointestinal toxicity. However, longer follow‐up is required to assess the efficacy of this treatment as well as its effect on biochemical control.

It is well known that the PLN are relatively fixed with respect to the pelvic vasculature, or the nearby bony anatomy, and move independently of the prostate. This raises the question of adequacy of PLN coverage when the image‐guided setup based on a prostate fiducial match is utilized for patients undergoing intensity modulated radiation therapy. This has been extensively studied for conventional fractionation, and it has been concluded that over a conventionally fractionated course of treatment, random shifts will provide adequate coverage that is unlikely to result in impactful underdosing.^9^, ^10^, ^11^, ^12^ However, limited studies with small number of patients exist regarding pelvic node coverage using SBRT.^13^ In this scenario, in addition to systematic errors, random errors may also have a large negative impact.^14^ The purpose of this study was to assess PLN coverage based on prostate fiducial matching during daily SBRT treatment.

## 2 MATERIALS AND METHODS

Thirty intact gland prostate patients who underwent five fraction SBRT treatment to the prostate and PLN from 2014‐2016 were evaluated in this IRB‐approved study. Each patient received either 800 cGy × 5 fx or 500 cGy × 5 fx to the prostate and 500 cGy × 5 fx to PLN. Pelvic CTV lymph node volumes were delineated up to aortic bifurcation by placing a 7 mm margin around the vessels, carving out bowel, bladder, and bone tissue, as per Radiation Therapy Oncology Group genitourinary radiation oncology specialists consensus.^15^ There are currently no guidelines for PLN hypofractionation, and the same margins around the vessels to generate clinical target volume (CTV) lymph nodes when moving from a conventional to a hypofractionation scenario have been used. Additionally, a 5 mm margin around the CTV was used to create the planning target volume (PTV) to account for day‐to‐day setup variation. Prostate CTV volume included entire prostate and bilateral seminal vesicles. A uniform 5 mm margin around the prostate CTV except a 3 mm margin at the prostate‐rectal interface was used to create the prostate PTV. All patients were treated on Varian^TM^ linear accelerator and positioned using the on‐board cone beam CTs (CBCT).

### 2.1 Simulation protocol

All 30 patients were simulated with a full bladder protocol (1 cup or 235 ml/45 min). Bowel prep included: Metamucil (1 tbsp/8 oz) for 7 days prior to simulation and throughout SBRT; a Fleet enema 3 h before simulation and daily treatments for SBRT; GasX the night before and the morning of simulation and daily treatments. Each patient underwent CT simulation in the supine position with a full bladder in a thermoplastic immobilization mold extending from the abdomen to mid‐thigh. CT simulation was followed by MR simulation on a 3 T scanner in the treatment position incorporating the patient's immobilization via the use of an indexed, flat tabletop. CT scans were acquired on a 16‐slice CT scanner (Philips Healthcare, Cleveland, OH) with 2 mm slice thickness extending from L1 to well below the ischial tuberosities. A Foley catheter and a rectal catheter (used only if there was gas in the rectum) were used for both CT and MR simulation. Three gold fiducial markers of 3 mm length and 1.2 mm diameter were implanted into the prostate under ultrasound guidance roughly 2 weeks prior to simulation. These markers were used to confirm and monitor the prostate position before and during each SBRT treatment using image guidance. During treatment, CBCTs were acquired for initial image‐guided setup. Each patient underwent five CBCT acquisitions. Daily CBCTs were also used to assess adequate bladder and rectum filling.

### 2.2 Image analysis

A total of 150 CBCTs (30 patients × 5CBCTs) were evaluated. Each CBCT had an in‐plane pixel resolution of 0.9 mm and a slice thickness of 2.0 mm. The longitudinal (superior‐inferior [SI]) extent was 16.0 cm which was sufficient to contour bladder volume for all patients except one where the bladder was much fuller during treatment and was missing a few superior slices. Two sets of registrations for each of the daily CBCTs were performed: a rigid registration to fiducials and a rigid registration to the bones (as a surrogate of PLN match). The registrations were performed in Eclipse^TM^ treatment planning system Version 13.6 (Varian Medical Systems, Inc.). Translational shifts were calculated by taking the difference between fiducial match and bony match. Although bony registration was automatic, fiducial match was manual. Rotations were not included when determining the translational shifts because this is the current clinical standard. A single experienced physician contoured rectal and bladder volumes on each daily CBCT to assess the variation from pretreatment volumes observed at simulation. Rectum volumes were consistently drawn from anal canal (inferiorly) up to recto‐sigmoid flexure (superiorly). The average translational difference between fiducial and bony match as well as the percentage of fractions with differences > 5 mm were calculated for the entire population. Changes in bladder and rectal volume with respect to the simulation volume and their correlation with translational shifts were evaluated by calculating pairwise correlation coefficients. In addition, the changes in COM of the bladder and rectal position from the pretreatment position, and their correlation with the translational shifts were also evaluated by calculating pairwise correlation coefficients. Coefficients with *P* < 0.05 were considered statistically significant. The coefficient values and the strength of association was calculated using general guidelines provided by Cohen et al.^16^ as shown in Table 1.

**Table 1:** Correlation coefficient values and the strength of association.^16^

Coefficient value	Strength of association
0.1 < |*r*| < 0.3	Small/weak correlation
0.3 < |*r*| < 0.5	Medium/moderate correlation
|*r*| > 0.5	Large/strong correlation

### 2.3 Dosimetric analysis

Dose calculations on all treatment plans were performed using Analytical Anisotropic Algorithm (AAA) in Eclipse treatment planning system Version 13.6. Two to three 15 MV arcs using volumetric modulated arc therapy technique (VMAT) was utilized. During planning, optimization was performed to achieve PLN PTV D95 ≥ 90% and PTV mean dose of 101%–103%. The dosimetric impact of the translational shifts on PLN coverage was evaluated by shifting the plan isocenter based on the translational shifts calculated between fiducial and bony match for each fraction. The beams were moved in the direction opposite to the direction of translational shift to simulate the patient shift. Once the beams were moved for each fraction on the planning CT, the dose was recalculated. The effect on CTV PLN D95 and CTV PLN V100 was evaluated. The dosimetric difference between planning and dose recalculation based on the translational shift was also calculated.

## 3 RESULTS

### 3.1 Percentage fractions with shifts > 5 mm

**Figure 1:**
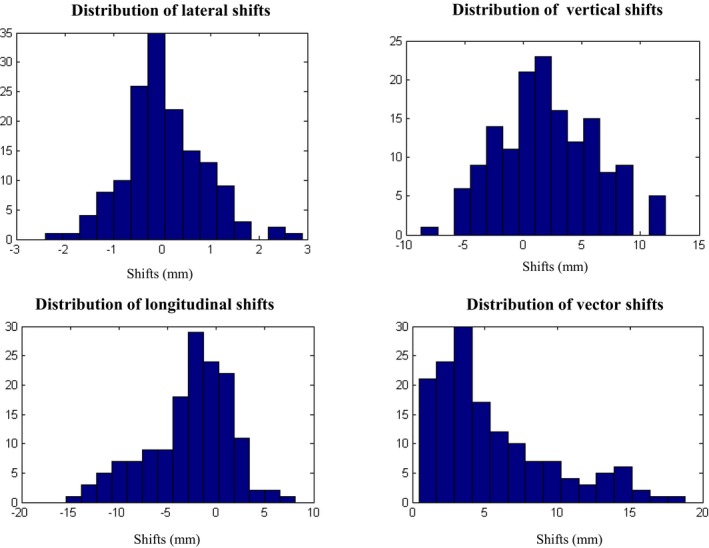
Distribution of lateral (X), vertical (Y), longitudinal (Z), and vector shifts between fiducial match and bony match for all patients and all fractions (30 patients × 5fx = 150 fxs).

A total of 150 CBCTs were analyzed in this study. The average translational difference between fiducial match and bony match was 0.06 ± 0.82, 2.1 ± 4.1, −2.8 ± 4.3, and 5.5 ± 4.2 mm along the lateral (left‐right [LR]), vertical (anterior‐posterior [AP]), longitudinal (superior‐inferior [SI]), and vector directions, respectively. The percentage of fractions with translational shifts > 5 mm were 0%, 25% (37/150), 23% (34/150), and 41% (61/150) for LR, AP, SI, and vector shifts, respectively. Seventeen out of 30 patients had shifts > 5mm in at least one direction on at least one fraction. Figure 1 shows the histogram distributions of LR, AP, SI, and vector shift for all 30 patients. Translational shifts along the LR direction were <3 mm for all fractions. The percentage of patients with translational shifts > 5mm along the SI direction in the first, second, third, fourth, and fifth fractions were 26.7%, 30%, 36.7%, 26.7%, and 23.3%, respectively. The percentage of patients with translational shifts > 5 mm along the AP direction in the first, second, third, fourth and fifth fractions were 30%, 26.7%, 26.7%, 16.8%, and 30%, respectively. The average percentage of fractions over all the treatment fractions with translational shifts > 5mm along the SI and AP directions were 28.7% and 26.04%, respectively.

### 3.2 Effect of changes in bladder and rectal volumes and their COM positions on the translational shifts

The average change in bladder volume from simulation was −67.2 ± 163.04 cc (−12 ± 52%). Pairwise correlation calculated between translational shifts and change in bladder volume was not statistically significant (see Table 2). The COM position of bladder was calculated for all the fractions. The average change in COM for the bladder from the simulation position was 0.34 ± 2.49, 4.4 ± 8.1, and −3.9 ± 7.5 mm along the LR, AP, and SI directions. Pairwise correlation between the translational shift and change in bladder COM position showed that AP translational shifts had a moderate correlation with the LR and AP change in COM position respectively and SI translational shift showed a weak to moderate correlation with LR, AP and SI change in COM position of the bladder.

**Table 2:** Pairwise correlation between translational shifts based on fiducial to bony match, and the percentage of change in bladder volume as well as bladder center‐of‐mass (COM) position from simulation

	LR	AP	SI
ΔVolume (cc)	0.04 (*P* = 0.64)	0.121 (*P* = 0.139)	0.058 (*P*= 0.484)
ΔVolume (%)	0.100 (*P* = 0.22)	0.06 (*P* = 0.408)	0.007 (*P* = 0.937)
ΔCOM_x (mm)	0.098 (*P* = 0.23)	**0.311** b ** (*P* = 0.0001)**	**−0.241** a ** (*P* = 0.003)**
ΔCOM_y (mm)	0.05 (*P* = 0.564)	**0.356** b ** (*P* < 0.0001)**	**−0.348** b ** (*P* < 0.0001)**
ΔCOM_z (mm)	0.137 (*P* = 0.09)	−0.123 (*P* = 0.133)	**0.239** a ** (*P* = 0.003)**

a Weak correlation.

b Moderate correlation.

^t^ Bold numbers are statistically significant.

**Figure 2:**
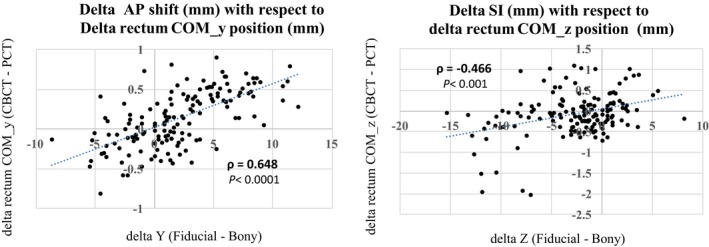
A scatter plot of vertical and longitudinal translational shifts (based on fiducial to bony match) with respect to vertical and longitudinal change in rectum center of mass position on CBCT from simulation position (Cone beam CT (CBCT) — Planning CT (PCT)) respectively for all patients and all fractions (30 patients × 5fx = 150 fxs).

The average change in rectal volume from simulation was −1.6 ± 18.75 (−2 ± 30%) cc. The average change in COM position from simulation position was 0.17 ± 1.9, 1.34 ± 3.5, and −0.6 ± 5.2 mm, respectively. Pairwise correlation between translational shifts and the change in rectum volume showed a negative, weak, statistically significant correlation with the AP translational shift and a positive, weak, statistically significant correlation with the SI translational shifts (see Table 3). Pairwise correlation between the translational shift and change in rectum COM position showed strong correlation between AP translational shift and change in AP COM position as well as moderate correlation between SI translational shift and AP and SI change in COM position. A weak correlation was observed between LR translational shift and LR change in COM position of rectum. Figure 2 shows scatter plots of vertical and longitudinal translational shifts with respect to vertical and longitudinal change in rectum center of mass position on CBCT from simulation position.

**Table 3:** Pairwise correlation between translational shifts based on fiducial to bony match and percentage of change in rectal volume as well as rectum center‐of‐mass (COM) position from simulation.

	LR	AP	SI
ΔVolume (cc)	0.119 (*P* = 0.149)	**−0.273** a ** (*P* = 0.0006)**	**0.193** a ** (*P* = 0.019)**
ΔVolume (%)	0.126 (*P* = 0.126)	**−0.272** a ** (*P* = 0.0008)**	**0.17** a ** (*P* = 0.039)**
ΔCOM_x (mm)	**0.286** a ** (*P* = 0.0004)**	−0.134 (*P* = 0.102)	0.098 (*P* = 0.236)
ΔCOM_y (mm)	0.035 (*P* = 0.670)	**0.648** c ** (*P* < 0.0001)**	**−0.466** b ** (*P* < 0.001)**
ΔCOM_z (mm)	−0.015 (*P* = 0.85)	−0.275 (*P* = 0.0007)	**0.362** b ** (*P* < 0.0001)**

a Weak correlation.

b Moderate correlation.

c Strong correlation.

^s^ Bold numbers are statistically significant.

### 3.3 Dosimetric impact

Mean CTV PLN D95 on the planning scan was 100% (range: 88.8%–108%). With the translational shifts, the average CTV PLN D95 coverage of the entire population for fractions one to five was 95.7% (range: 65%–105%), 96.8% (range: 70.3–105.7%), 96% (range: 60.0–105.7%), 97.6% (range: 69.2%–105.7%), and 96.0% (range: 70.2%–105.7%), respectively. The percentage of fractions with CTV PLN D95 < 95% for fractions 1 to 5 was 27%, 27%, 27%, 17%, and 27%, respectively. The percentage of fractions with CTV PLN D95 < 90% was 14%, 14%, 14%, 10%, and 17%, respectively. The average percentage of fractions over all treatment fractions with CTV PLN D95 < 95% and CTV PLN D95 < 90% were 25% and 13.8%, respectively. Only two patients had CTV PLN D95 < 90% for all five fractions, three patients with CTV PLN D95 < 90% in four fractions, one patient with CTV PLN D95 < 90% in three fractions and three patients with CTV PLN D95 < 90% in one fraction. One patient had CTV PLN D95 of ~ 89% during planning. This patient had D95 < 90% for all five fractions. Seven patients had average CTV PLN D95 < 90% for all five fractions.

Mean CTV PLN V100 on the planning scan was 96.7% (range: 79%–100%). With the translational shifts, the average CTV PLN V100 coverage of the entire population for fractions one to five was 91.5% (range: 59.2%–100%), 92.2% (range: 68.3–100%), 91.4% (range: 51.0–100%), 93.0% (range: 67.1%–100%), and 91.3% (range: 47%–100%), respectively. Nine patients had average CTV PLN V100 < 90% for all five fractions. Poor dosimetric coverage was associated with larger translational shift. A pairwise correlation between translational shift and CTV PLN D95 and V100 showed a strong negative correlation with the AP translational shift (*P* < 0.0001) and strong positive correlation with the SI translational shift (*P* < 0.0001). (Table 4).

**Table 4:** Pairwise correlation between translational shifts based on fiducial to bony match, and clinical target volume (CTV) dosimetric parameters D95 and V100.

	LR	AP	SI
CTV PLN D95	−0.069 (*P* = 0.40)	**−0.667** a **(*P* < 0.00001)**	**0.6998** a **(*P* < 0.0001)**
CTV PLN V100	−0.082 (*P* = 0.32)	**−0.666** a **(*P* < 0.00001)**	**0.6139** a **(*P* < 0.0001)**
CTV PLN Δd95	−0.156 (*P* = 0.1983)	**−0.7120** a **(*P* < 0.0000)**	**0.7570** a **(*P* < 0.0000)**
CTV PLN ΔV100	−0.1591 (*P* = 0.0518)	**−0.6753** a **(*P* < 0.0000)**	**0.6736** a **(*P* < 0.0000)**

a Strong correlation.

^q^ Bold numbers are statistically significant.

The dosimetric difference between planning and dose recalculation based on the translational shift was also calculated and correlated with the translational shifts. Similar to the absolute dose metric, change in dosimetric parameter also showed a strong negative correlation with the AP translational shift and strong positive correlation with the SI translational shift.

## 4 DISCUSSION

In this study, we examined the effect of soft tissue‐based daily image guidance on PLN coverage of prostate cancer patients undergoing SBRT. This is one of the first studies to evaluate 30 prostate patients undergoing SBRT with PLN irradiation and daily CBCT‐guided fiducial‐based image guidance. Our data indicate that up to 75% of fractions were covered within 5 mm margin near the PLN when they were positioned daily based on fiducials in the prostate. A 7 mm margin would cover approximately 90% of the patient population translational shifts along the SI and AP directions were more significant than those along the LR direction. Shifts up to 15 mm were seen in the SI and AP directions. Some of these larger shifts were due to large prostate rotations, or large variation in bladder and rectal volumes. The current analysis does not take into account the prostate rotation because this is not the current clinical practice at our institution.

In terms of dosimetric impact of these translational shifts, we found, on average, ~19% of the fractions had CTV D95 coverage of <90% as a result of these shifts. One patient had CTV PLN D95 < 90% during planning as well as during all subsequent fractions. Excluding this patient, ~16% of the fractions had CTV D95 coverage of <90% as a result of these shifts. PLN coverage based on prostate fiducial match for intermediate‐ to high‐risk prostate cancer patients has been extensively studied for conventional fractionation. Hsu, et al. reported a <1.5% difference in dose delivered to the PLN for five patients with simulated random shifts, and a 10% difference in dose delivered to the PLN, modeling a 1 cm systematic displacement using a 5 mm planning margin around the lymph nodes.^10^ Only one study looked at the dosimetric impact of PLN coverage in a SBRT setting based on 5 mm CTV‐to‐PTV margin. Kishen, et al. analyzed 12 patients (65 CBCT scans) undergoing SBRT with PLN irradiation and found the average V100 CTVN was 92.6%, but for a subset of three patients, the average was 80.0%, compared with 97.8% for the others [*P* < 0.0001]).^13^ These patients had large bone‐to‐fiducial translational shifts and a large variation in bladder height (calculated on the anteriormost coronal plane). Six out of 30 patients in our study had an average CTV D95 < 90% (79%, 85%, 86%, 76%, 82%, 89%, respectively averaged over all five fractions). Eight patients including the six above had CTV PLN V100 < 90%. Three of these patients had their CTV PLN V100 < 90% in the reference treatment plan as well. Our study looked at changes in bladder volume and bladder COM position instead of bladder height to investigate correlation with the translational shifts. Although change in bladder volume showed no correlation, change in COM position of bladder showed a weak to moderate correlation with the translational shifts. COM position is a more widely accepted metric compared bladder height. As compared with Kishen, et al., where PLNs were contoured on the CBCTs and the dose was recalculated, the SI extent of CBCT in our study was only approximately 16 cm, and the full extent of the PLNs could not be contoured on the CBCT to assess their dosimetric coverage based on soft tissue match. Instead, the isocenter shifting technique was used to simulate the effect of these translational shifts on the planning CT.

Daily variations in rectal volume as well as changes in COM position of the bladder and rectum were correlated with the translational shifts. Our bowel prep protocol includes using GasX/enema the night before and on the day of the treatment. Patients are also instructed to drink one cup (or ~235 ml)/45 min, but patient queue and machine delays may add to the variation in bladder filling. The bladder and rectal volumes were also systematically smaller than the simulation volume for the majority of patients. Although a much larger variation in bladder volume was observed compared with rectal volume, the change in bladder volume was not correlated with the translational shifts. We believe changes in bladder volume might push the bladder anteriorly and superiorly but with a lesser effect on the actual prostate. The change in COM of rectum was more strongly correlated with translation shifts as compared to change in COM of bladder.

Finally, our study supports the inclusion of PLN in CBCT scans during daily image guidance for SBRT cases by shifting the couch longitudinally by approximately 5 cm. Physicians reviewing the daily CBCT scans should also look at PLN coverage in addition to prostate coverage, and if a large discrepancy is observed, it should be investigated before proceeding with the treatments.

## 5 CONCLUSIONS

Our study indicates that a 5 mm margin provides coverage for ~75% of patients receiving PLN irradiation with SBRT, daily CBCT and fiducial‐guided setup. In 19% of fractions or 25% of patients, the dosimetric impact on PLN coverage was significant. The largest translational shifts were seen in the vertical and longitudinal directions and were due to variation in rectal volume as well as changes in COM position of the bladder. This indicates that consistent bladder positioning and/or rectum filling compared with volumes at simulation is essential for adequate coverage of PLN in a hypofractionated treatment regime.

## CONFLICT OF INTERESTS

None.
